# Aberrant Functional Connectivity of the Amygdala Complexes in PTSD during Conscious and Subconscious Processing of Trauma-Related Stimuli

**DOI:** 10.1371/journal.pone.0163097

**Published:** 2016-09-15

**Authors:** Daniela Rabellino, Maria Densmore, Paul A. Frewen, Jean Théberge, Margaret C. McKinnon, Ruth A. Lanius

**Affiliations:** 1 Department of Psychiatry, University of Western Ontario, London, ON, Canada; 2 Imaging Division, Lawson Health Research Institute, London, ON, Canada; 3 Department of Psychology, University of Western Ontario, London, ON, Canada; 4 Department of Neuroscience, University of Western Ontario, London, ON, Canada; 5 Department of Medical Biophysics, University of Western Ontario, London, ON, Canada; 6 Mood Disorders Program, St. Joseph's Healthcare, Hamilton, ON, Canada; 7 Department of Psychiatry and Behavioural Neurosciences, McMaster University, Hamilton, ON, Canada; 8 Homewood Research Institute, Guelph, ON, Canada; Yale University, UNITED STATES

## Abstract

Post-traumatic stress disorder (PTSD) is characterized by altered functional connectivity of the amygdala complexes at rest. However, amygdala complex connectivity during conscious and subconscious threat processing remains to be elucidated. Here, we investigate specific connectivity of the centromedial amygdala (CMA) and basolateral amygdala (BLA) during conscious and subconscious processing of trauma-related words among individuals with PTSD (*n* = 26) as compared to non-trauma-exposed controls (*n* = 20). Psycho-physiological interaction analyses were performed using the right and left amygdala complexes as regions of interest during conscious and subconscious trauma word processing. These analyses revealed a differential, context-dependent responses by each amygdala seed during trauma processing in PTSD. Specifically, relative to controls, during subconscious processing, individuals with PTSD demonstrated increased connectivity of the CMA with the superior frontal gyrus, accompanied by a pattern of decreased connectivity between the BLA and the superior colliculus. During conscious processing, relative to controls, individuals with PTSD showed increased connectivity between the CMA and the pulvinar. These findings demonstrate alterations in amygdala subregion functional connectivity in PTSD and highlight the disruption of the innate alarm network during both conscious and subconscious trauma processing in this disorder.

## Introduction

Altered threat processing and alerting mechanisms are central to the pathophysiology of posttraumatic stress disorder (PTSD). Previous studies investigating the neural correlates underlying threat responses have emphasized functional connectivity alterations between the amygdala and the medial prefrontal cortex (mPFC) during symptom provocation [1], fear processing [2] and at rest [3], suggesting aberrant emotion regulatory capacity in PTSD. Moreover, the functional connectivity of the amygdala with the insula, a neural region associated with consciousness and self-awareness, appears altered in PTSD, showing differential context-dependent results. Here, recent studies revealed increased connectivity between these regions during resting state [4,5] that contrasts with decreased connectivity during threat-related processing [6].

One key limitation of the majority of previous studies examining amygdala connectivity in PTSD subjects is the limited analysis of the amygdala as whole, despite knowledge that the amygdala complex is composed of distinct subdivisions, including the centromedial amygdala (CMA) and the basolateral amygdala (BLA) nuclei that are known to have diverse functions and differential functional connectivity with other brain regions [7–9]. The CMA, including the central and medial nuclei, contains GABAergic neurons [10] and, through increased attention and motor readiness, facilitates behavioral responses to emotion via projections to the brainstem, hypothalamus (regulating cortisol release), basal forebrain [11], and striatal regions [8,12,13]. By contrast, the BLA comprises the lateral, basolateral, basomedial, and basoventral nuclei and through its thalamic projections functions to integrate sensory inputs with cortical association areas [14] in order to facilitate learning (i.e., fear conditioning; [7,9]). In one preliminary study informed by emerging evidence for the distinct functional roles of the CMA and BLA in threat processing [15–17], Brown *et al*. (2013) [7] showed increased connectivity between the BLA and the prefrontal cortical regions involved in emotion regulation in PTSD subjects as compared to trauma-exposed controls at rest. Moreover, dissociative (depersonalization and derealization) symptoms in PTSD correlated with increased connectivity between both the CMA and the BLA with prefrontal regions and with regions involved in body awareness and proprioception at rest (precuneus and dorsal posterior cingulum) [17]. Despite these provocative findings, the functional connectivity of amygdala subregions during exposure to trauma-related stimuli at the conscious and subconscious level has yet to be fully explored.

The present study seeks to address this central gap in our understanding of threat-related processing in PTSD. PTSD patients are often triggered by stimuli of which they have no conscious awareness, where altered patterns of neural activity underlying subconscious processing of fearful or trauma-related material are thought to contribute to this symptom presentation [18–21]. Here, aberrant alerting mechanisms in PTSD have been associated with altered neural activity within key regions of the innate alarm system (a network of areas involved in rapid alerting to threat, including the brainstem, amygdala, pulvinar, and mPFC, see [22]), and increased brainstem-amygdala connectivity [20,23–26]. To date, only two studies have investigated functional connectivity during subconscious threat processing in PTSD, revealing aberrant amygdala functional connectivity within the default mode network (comprising prefrontal and posterior regions [27]) and altered amygdala-mPFC connectivity [24]. Strikingly, Bryant and colleagues (2008) [24] explored whole amygdala functional connectivity during subconscious fear processing and found differential interhemispheric effects, with increased right amygdala-left mPFC coupling and decreased left amygdala-right mPFC coupling when comparing PTSD subjects to controls. Taken together, these findings reviewed here point towards the need for further investigation of functional connectivity of the CMA and BLA at both the conscious and subconscious levels of awareness.

Accordingly, the present study examines the differential contribution of subdivisions to brain connectivity during conscious and subconscious processing of personalized trauma-related words in PTSD. In line with emerging reports, we expected to find differential functional connectivity between the BLA and CMA with the mPFC, parietal and insular cortex as a function of conscious and subconscious levels of awareness in PTSD. Finally, we sought to define the role of each specific amygdala subregion within the innate alarm circuit in PTSD, particularly with reference to brainstem-amygdala connectivity [26] during subconscious and conscious threat processing.

## Methods

### Participants

Twenty-six civilians with a primary diagnosis of PTSD and twenty non trauma-exposed controls were recruited through community advertisement to participate in the study. This sample has been described in two previous studies [27,28]. All participants were administered the Clinician Administered PTSD Scale (CAPS) to diagnose PTSD (cut-off score > 50) [29], the Childhood Trauma Questionnaire–Short Form (CTQ) [30], the Structured Clinical Interview for DSM-IV Axis I disorders (SCID-I) [31], and the Multiscale Dissociative Inventory (MDI) [32]. Whereas twenty-three out of 26 individuals (88.46%) with PTSD had experienced childhood interpersonal trauma, 3 out of 26 (11.54%) individuals with PTSD had experienced a personal life threat or were witnesses to a violent death. Demographic and psychological characteritics are summarized in Table 1. Exclusion criteria were bipolar disorder, a lifetime diagnosis of psychosis, substance or alcohol use disorder within the last six months, serious medical conditions or neurologic illness, a history of head injury with a loss of consciousness, and fMRI incompatibility. All participants were right handed. Controls did not meet any current or lifetime criteria for psychiatric disorders, and PTSD patients were medication free for at least six weeks prior to participating in the study. Approval was obtained from the Health Sciences Research Ethics Board of Western University, Canada, and all participants received a detailed description of the study protocols and provided informed written consent.

**Table 1:** Clinical and demographic information divided by group.

Clinical and demographical characteristics	PTSD group (n = 26)	Comparison group (n = 20)	t-test/χ2 (*p*)
Age (mean ± SD) years	38.79 ± 12.17	32.5 ± 11.58	0.088
Gender (F) frequency	15	10	0.604
Employed frequency	18	17	0.297
CAPS tot score (mean ± SD)	70.57 ± 11.86	0.94 ± 2.91	< 0.001**
MDI tot score (mean ± SD)	59.96 ± 21.26	33.7 ± 3.79	< 0.001**
CTQ Emotional abuse score (mean ± SD)	14.48 ± 6.13	6.75 ± 3.09	< 0.001**
CTQ Physical abuse score (mean ± SD)	10.08 ± 6.39	5.65 ± 1.59	0.004**
CTQ Sexual abuse score (mean ± SD)	13.44 ± 7.75	5.25 ± 1.12	< 0.001**
CTQ Emotional neglect score (mean ± SD)	13.52 ± 5.92	8.8 ± 4.17	0.004**
CTQ Physical neglect score (mean ± SD)	10.24 ± 4.70	6.8 ± 2.72	0.006**
AXIS I comorbidity (current [past]) frequency	Major depressive disorder (8 [9])	-	-
	Dysthymic disorder (0 [3])		
	Panic disorder with agoraphobia (0[1])		
	Panic disorder without agoraphobia (1[1])		
	Agoraphobia without panic disorder (3)		
	Social phobia (4)		
	Specific phobia (2)		
	Obsessive-compulsive disorder (1[1])		
	Eating disorders (1[1])		
	Somatoform disorder (6)		
	Lifetime history of alcohol abuse or dependence [16]		
	Lifetime history of substance abuse or dependence [7]		

CAPS, Clinical Administered PTSD Scale; CTQ, Child Trauma Questionnaire; MDI, Multiscale Dissociation Inventory; PTSD, Post-Traumatic Stress Disorder

** *p*< .01

### FMRI Protocol

**Fig 1:**
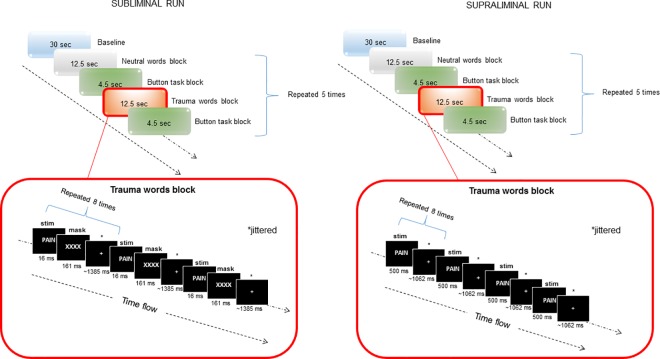
Graphical depiction of the experimental design. **Subliminal session is depicted on the left, supraliminal session on the right. Above panels depict the block design. Below panels depict timing windows and stimulus presentation within word blocks (the trauma-related word block in this case).** Note: ms: milliseconds; sec: seconds; stim: stimulus.

The protocol used for presenting subconscious (subliminal) and conscious (supraliminal) stimuli during fMRI investigations followed previously published methods [27,33,34]. Briefly, stimuli included personalized trauma-related and neutral words provided by the participants during the assessment stage. Whereas trauma-related stimuli referred to words subjectively perceived as direct cues for trauma in PTSD patients (or for a stressful experience in controls), neutral cues were words that did not elicit any strong reaction (either positive or negative). All words were matched for letter/syllable length (examples of words used is available in S1 Table). A block design was used, which included five blocks for each stimulus. Each block consisted of eight repetitions of the stimulus separated by jittered inter-stimulus intervals (823–1823 msec in the subliminal condition, 500–1500 msec during the supraliminal condition). All stimuli were presented both subliminally (16 msec backward-masked for 161 msec; [22,35–40]) and supraliminally (500 msec; [33,34,39]) over two consecutive sessions, respectively, counterbalanced between-subjects (see Fig 1 for a graphical depiction of the experimental design). A 2-minute rest period separated the two sessions. Participants were instructed to view passively the stimuli presented using E-Prime software 2.0 (2007, Psychology Software Tools) and displayed via an external video projector (Sharp DLP XG-PH70X-N) and a mirror system. Stimuli duration was verified using a PIN diode with a 40 kHz A/D converter. A button press task (letter recognition; 4500 msec) was added between blocks, in order to control for continued attention of participants, and a 30-second rest period at the beginning of each run was used as an implicit baseline for subsequent analyses.

### FMRI data acquisition

A 3.0 Tesla whole-body MRI scanner (Magnetom Tim Trio, Siemens Medical Solutions, Erlangen, Germany) with a 32-channel phased array head coil was used for image acquisition. T1-weighted anatomical images were acquired with 1 mm isotropic resolution (MP-RAGE, TR / TE / TI = 2300 ms / 2.98 ms / 900 ms, flip angle = 9 degrees, FOV = 256 mm x 240 mm x 192 mm, acceleration factor = 4, total acquisition time = 3 min 12 s). Sixty four whole-brain, 2 mm thick imaging planes for BOLD fMRI were prescribed parallel to the AC-PC. Functional images were acquired using a gradient-echo planar imaging (EPI) sequence with an interleaved slice excitation order and a 2 mm isotropic spatial resolution (FOV = 192 mm x 192 mm, 94 x 94 matrix, TR / TE = 3000 ms / 20 ms, flip angle = 90 degree, acceleration factor = 4, 24 reference lines, 64 slices, 250 volumes).

### Statistical Analyses

#### Participants' characteristics

Independent sample t-tests were used to investigate between-group differences in age, CTQ score, MDI score, and reaction time during the button-press task. Pearson's chi-square tests were performed for gender and employment status.

#### FMRI analyses

FMRI analyses were performed using *SPM8* (Wellcome Trust Centre for Neuroimaging, London, UK) implemented in *MATLAB* (R2013a; Mathworks Inc., Sherborn, MA, USA). Pre-processing included realignment to the first image, coregistration of the anatomical image to the mean functional image, normalization to MNI (Montreal Neurological Institute) space (spatial resolution 2 x 2 x 2), and spatial smoothing with an 8 mm full width at half maximum (FWHM) isotropic Gaussian Kernel.

In order to investigate trauma-related effective functional connectivity, we conducted psychophysiological interactions (PPI) analyses [41]. PPI represents a reliable technique to examine how neural activity in a specified seed region influences the activity in another area as a function of the experimental context.

To investigate effective connectivity between the amygdala and brain areas involved in threat processing, we defined four seed regions using SPM Anatomy toolbox [42,43], including the left and right centromedial amygdala (CMA) and the left and right basolateral amygdala (BLA). The centromedial amygdala (CMA) included the central nucleus and the medial nucleus (volume left CMA = 138±31mm^3^, volume right CMA = 138±28mm^3^). By contrast, the basolateral amygdala (BLA) encompassed the lateral nucleus, the basolateral, basomedial and paralaminar nuclei (left BLA volume = 1063±214 mm^3^, right BLA volume = 1050±219mm^3^). For each seed region we extracted the first eigenvariate of the BOLD time-series in the GLM derived contrasts subliminal trauma-related > neutral words and supraliminal trauma-related > neutral words for each subject. The PPI interaction terms were obtained by multiplying the deconvolved time-series by the psychological variable (subliminal or supraliminal trauma-related > neutral word condition) and reconvolved with the hemodynamic response function (HRF). The motion parameters were included as confounds in the design matrix. The estimated interaction term parameters were then carried forward to the second level to perform one-sample (within-group) and two-sample t-tests (between-groups) for each seed-region.

Whole brain analyses (voxelwise p < .05 FWE-corrected threshold) were followed by Region of Interest (ROI) analyses. Here, three10 mm sphere radius ROIs were tested based on previous literature examining subliminal fear processing [22] and functional connectivity of amygdala subdivisions in PTSD [7] in order to investigate the brainstem-amygdala-cortical innate alarm circuitry involved in sub- and supraliminal threat processing [bilateral superior colliculus (SC; centered in -0.5, -21, -8, MNI; [22]), left pulvinar (centered in -16, -24, 10, MNI; [22]); and right medial prefrontal cortex (centered in 14, 42, 42, MNI; [7]). All results were considered significant when meeting the voxelwise p < .05 FWE-corrected threshold within each ROI, adjusted for multiple comparisons (comprising 4 seed regions and 3 ROIs, yielding p < .004 FWE-corrected).

## Results

### Participants

As has been reported previously [27], no significant differences emerged between PTSD participants and controls on any demographic measure as well as on reaction times relative to the button-press task. However, as expected, the PTSD group scored significantly higher on the CAPS total, the CTQ scales, and the MDI total (see Table 1 for details) as compared to the control group.

### Effective connectivity in PTSD as compared to controls during trauma processing

#### Subliminal (subconscious) processing

**Fig 2:**
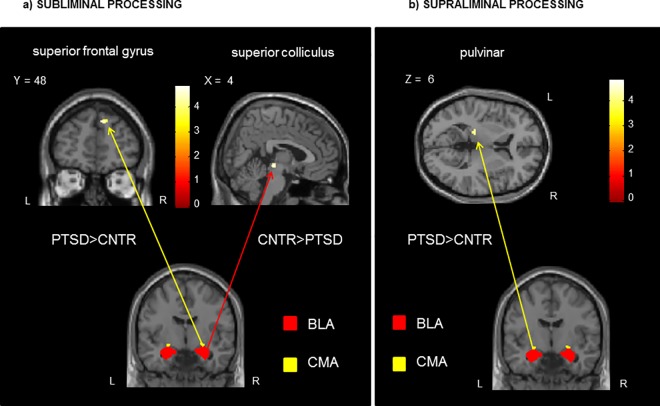
**On the left, a) Increased (PTSD>CNTR) and decreased (CNTR>PTSD) functional connectivity of the amygdala complexes during processing of SUBLIMINAL (subconscious) trauma-related words in PTSD as compared to controls. On the right, b) Increased (PTSD>CNTR) functional connectivity of the amygdala complexes during processing of SUPRALIMINAL (conscious) trauma-related words in PTSD as compared to controls. Coordinates are reported in MNI. Color bar indicates *t* scores.** Note: BLA: basolateral amygdala; CMA: centromedial amygdala; CNTR: control group; L: left; PTSD: post-traumatic stress disorder group; R: right.

Whole-brain analyses did not yield any significant results. By contrast, ROI analyses showed increased connectivity between the right CMA and the superior frontal gyrus (SFG) and decreased connectivity between the right BLA and the right superior colliculus in the PTSD as compared to the control group. (Table 2; Fig 2A).

**Table 2:** Functional connectivity of each amygdala subdivision during sub- and supraliminal trauma-related word processing.

Condition/seed	Contrast	Brain regions	Hemisphere	MNI coordinates			*k*	peak *z* score
				x	y	z		
**Subliminal Trauma>Neutral Words**								
Right centromedial	PTSD>CNTR	superior frontal gyrus	R	12	50	38	21	4.15
Right basolateral	CNTR>PTSD	superior colliculus	R	4	-26	-6	22	4.00
**Supraliminal Trauma>Neutral Words**								
Left centromedial	PTSD>CNTR	pulvinar	L	-18	-28	6	16	3.95

Note: all results are reported with voxelwise FWE-corrected threshold of *p* < .05, adjusted for multiple comparisons, within *a priori* identified ROIs. CNTR: control group; *k*: cluster extent; PTSD: post-traumatic stress disorder group.

#### Supraliminal (conscious) processing

No significant results emerged from whole-brain analyses. However, ROI analyses revealed that the PTSD group showed increased connectivity between the left CMA and the left pulvinar in comparison to controls (Table 2; Fig 2B).

## Discussion

The results of the present study demonstrate clearly the contrasting roles played by the amygdala subregions investigated (the CMA and the BLA) in threat processing. Here, we found differential patterns of functional connectivity of these regions with cortical and subcortical regions during trauma-related word processing in PTSD subjects as compared to controls. Critically, these patterns of functional connectivity appeared to be moderated by the level of awareness of the threatening cues. Moreover, subcortical and cortical regions including the innate alarm network (i.e., superior colliculus, pulvinar, and mPFC) emerged as differentially functionally connected to the amygdala subdivisions. As this study did not include a trauma-exposed control group, it is important to bear in mind that the findings discussed below could also be interpreted as a result of trauma-exposure instead of specifically relating to the development of PTSD (see also Limitations section).

### Subliminal (subconscious) processing of trauma-related words in PTSD as compared to healthy controls

During subliminal processing, the right CMA showed increased connectivity with frontal regions—specifically with the medial SFG. It is therefore probable that this pattern of right CMA connectivity underlies Bryant and colleagues' (2008) [24] previous findings of increased coupling of the right amygdala with the mPFC during unconscious processing among PTSD participants. Notably, Bryant and colleagues (2008) [24] suggested the amygdala has an overly excitatory influence on the mPFC in PTSD, thus contributing to dysregulation of the fear network. In addition, previous studies investigating white matter integrity in individuals with PTSD suggested that diffusion alterations in the anatomical structures of prefrontal regions in PTSD would accompany functional abnormalities, accounting for emotion under-modulation and exaggerated threat reaction [44–46]. Our findings further support these suggestions.

It is also noteworthy that decreased functional connectivity of the BLA with the superior colliculus (SC) was identified in PTSD subjects. Whereas the BLA is the primary site of sensory input into the amygdala, allowing for affective evaluations of sensory information [9,14], the SC is a midbrain structure found to integrate multisensory information. Hence, decreased BLA-SC connectivity in PTSD would suggest a disruption of the integration between sensory inputs and their affective evaluations, possibly disturbing learning processes (such as fear conditioning). Alternatively, this finding may be related to the control function carried out by the SC in target selection for orienting quick behavioral responses (for a review see [47]). Here, disrupted BLA-SC functional connectivity in PTSD points towards the SC as a crucial node for immediate defensive responses occurring independent of higher-order emotion regulatory processes involving the amygdala and mPFC, as exemplified by autonomic responses including states of freezing or tonic immobility following threat [48]. Here, the individual would show a fast defensive reaction (i.e., freezing or tonic immobility and heightened vigilance) to threatening stimuli without the need to resort to slower higher-order emotion regulatory processes [49]. Both interpretations of BLA-SC connectivity, however, suggest altered innate alarm network responding to threat in PTSD.

In summary, our findings highlight the differential functions of amygdala subregions in PTSD during subliminal processing of threat. Specifically, increased functional connectivity of the CMA with the SFG during subconscious threat processing suggests the involvement of the CMA in emotion regulatory processes. By contrast, the decreased connectivity of the BLA with the superior colliculus in PTSD as compared to controls may contribute to disturbances in fear learning processes, but may also represent a neural mechanism for quick defensive responses occurring independently from regulatory limbic (amygdala) and cortical (cingulum and prefrontal cortex) regions (i.e., during fast responses to threat) in PTSD.

### Supraliminal (conscious) processing of trauma-related words in PTSD as compared to healthy controls

During supraliminal processing, the left CMA showed increased connectivity with the pulvinar, a subcortical region that is a key-node, in connection with the superior colliculus and the amygdala, of the innate alarm system for quick defensive responses to potential threats [22] and salience detection [50]. This finding suggests increased readiness in organizing behavioral responses during the detection of salient cues in PTSD subjects as compared to controls during supraliminal processing of threat-related stimuli.

## Limitations and Conclusions

Certain limitations of our study must be considered. Firstly, the present findings are based on a small sample size; replication with a larger sample is therefore needed. Secondly, our study did not include a trauma-exposed group without PTSD; future research should also compare trauma-exposed and PTSD groups. Moreover, it will be important to examine potential differences between PTSD and its dissociative subtype; the present study did not afford enough power to examine different phenotypes of the disorder. Finally, the cross-sectional nature of this study precludes assumptions about whether the findings are a consequence of PTSD; future studies are warranted to consider a longitudinal approach to address this matter.

These points notwithstanding, we conclude that our results provide further insight into the pathways modulated by the amygdala within the innate alarm system in PTSD. As highlighted by recent literature, the amygdala can be better understood if subdivided into distinct nuclei in order to show the differential involvement of each subregion in the alerting system for quick defensive responses. Moreover, our study design allowed us to distinguish which brain regions and hemispheres are predominant during sub- versus supraliminal threat processing. During subliminal processing, the right CMA was shown to play a major role in influencing neural activity in the prefrontal regions (SFG) as a function of threat processing. By contrast, supraliminal processing revealed that the left CMA is involved in modulating the subcortical pathway (i.e., the pulvinar) for defensive responses to potential threats. Furthermore, our findings clarify the significance of amygdala connectivity in PTSD, specifying, for example, which amygdala subregions are involved in amygdala-prefrontal connectivity during subliminal processing and how levels of stimulus awareness differentially influence the functional connectivity of the amygdala to other brain regions. Moreover, our findings support a right lateralization of a fast subcortical route for rapid defensive responses to subliminal threat [51,52]. Finally, we also observed disrupted amygdala- superior colliculus functional connectivity in PTSD as compared to controls that may be related to an alteration of the innate alarm network in fast defensive responses occurring at a subconscious level.

## Supporting Information

S1 Table Example of personalized trauma-related words and neutral words. Example of personalized trauma-related (stress-related for controls) words and neutral words used for stimulus presentation during the task in the fMRI scanner. As shown in the table, trauma-related and neutral words were matched for letter/syllable length.
(DOCX)
