# Supplementary material for: Aberrant Functional Connectivity of the Amygdala Complexes in PTSD during Conscious and Subconscious Processing of Trauma-Related Stimuli
Source: PLoS One. 2016 Sep 15;11(9):e0163097. doi: 10.1371/journal.pone.0163097 (PMC5025207; doi:10.1371/journal.pone.0163097)
Supplement: S1 Table — Example of personalized trauma-related (stress-related for controls) words and neutral words used for stimulus presentation during the task in the fMRI scanner. As shown in the table, trauma-related and neutral words were matched for letter/syllable length. (DOCX) [file pone.0163097.s001.docx]

**Supporting Information**

**S1 Table. Example of personalized trauma-related words and neutral words.** Example of personalized trauma-related (stress-related for controls) words and neutral words used for stimulus presentation during the task in the fMRI scanner. As shown in the table, trauma-related and neutral words were matched for letter/syllable length.

|  | **TRAUMA WORD** | **NEUTRAL WORD** |
| --- | --- | --- |
| **PTSD GROUP** | KNIFE | CHALK |
|  | RMYC | ASAP |
|  | LIAR | FROG |
|  | ABUSIVE | NEUTRAL |
|  | DISMISS | RELEASE |
|  | FACE | COMB |
|  | HIDE | WALK |
|  | SEX | SEE |
|  | PRIEST | STONES |
|  | PARENTS | PENCILS |
| **CONTROL GROUP** | DEATH | CHAIR |
|  | BRONZE | ERASER |
|  | DAD | AIR |
|  | MCAT | LSAT |
|  | FAT | HAT |
|  | SPIDERS | ORANGES |
|  | WASP | LAMP |
|  | CANCER | BOTTLE |
|  | HEART | LIGHT |
|  | AYLMER | PENCIL |

Legend: PTSD: post-traumatic stress disorder.
